# Social Determinants of Diabetes-Related Preventable Hospitalization in Taiwan: A Spatial Analysis

**DOI:** 10.3390/ijerph18042146

**Published:** 2021-02-22

**Authors:** Chung-Yi Li, Yung-Chung Chuang, Pei-Chun Chen, Michael S. Chen, Miaw-Chwen Lee, Li-Jung Elizabeth Ku, Chiachi Bonnie Lee

**Affiliations:** 1 Department of Public Health, College of Medicine, National Cheng Kung University, Tainan 701401, Taiwan; cyli99@mail.ncku.edu.tw (C.-Y.L.); eljku@mail.ncku.edu.tw (L.-J.E.K.); 2 Department of Urban Planning and Spatial Information, Feng Chia University, Taichung 407802, Taiwan; yungcchuang@fcu.edu.tw; 3 Department of Public Health, China Medical University, Taichung 40402, Taiwan; peichun@mail.cmu.edu.tw; 4 Department of Healthcare Administration, Asia University, Taichung 41354, Taiwan; hermitindependence@gmail.com; 5 Department of Social Welfare and Center for Innovative Research on Aging Society, National Chung Cheng University, Chiayi 62102, Taiwan; mclee137@ccu.edu.tw; 6 Department of Health Services Administration, China Medical University, Taichung 40402, Taiwan

**Keywords:** diabetes-related preventable hospitalization, social determinants of health, spatial analysis, Taiwan

## Abstract

Diabetes-Related Preventable Hospitalization (DRPH) has been identified as an important indicator of efficiency and quality of the health system and can be modified by social determinants. However, the spatial disparities, clustering, and relationships between DRPH and social determinants have rarely been investigated. Accordingly, this study examined the association of DRPH with area deprivation, densities of certificated diabetes health-promoting clinics (DHPC) and hospitals (DHPH), and the presence of elderly social services (ESS) using both statistical and spatial analyses. Data were obtained from the 2010–2016 National Health Insurance Research Database (NHIRD) and government open data. Township-level ordinary least squares (OSL) and geographically weighted regression (GWR) were conducted. DRPH rates were found to be negatively associated with densities of DHPC (β = −66.36, *p* = 0.029; 40.3% of all townships) and ESS (β = −1.85, *p* = 0.027; 28.4% of all townships) but positively associated with area deprivation (β = 2.96, *p* = 0.002; 25.6% of all townships) in both OLS and GWR models. Significant relationships were found in varying areas in the GWR model. DRPH rates are high in townships of Taiwan that have lower DHPC densities, lower ESS densities, and greater socioeconomic deprivation. Spatial analysis could identify areas of concern for potential intervention.

## 1. Introduction

The total health expenditure for adults (20–79 years) with diabetes has been growing considerably worldwide from US$ 232 billion in 2007 to US$ 760 billion in 2019, representing an increase of 328% [1]. In Taiwan, from 2005 to 2014, the total number of patients with diabetes rose by 66%, age-standardized prevalence in patients with diabetes aged 20–79 years rose by 41%, and diabetes incidence rose by 19% [2]. The average yearly costs per diabetes patient incurred at the inpatient department were 1.4 times higher than the general population, and the annual average medical cost of each diabetes patient with both micro-and macro-vascular complications was four times higher than those without such complications in Taiwan [3]. Diabetes-Related Preventable Hospitalization (DRPH) has been identified as an important indicator of efficiency and quality of the health system and has been adopted in the OECD Health Care Quality Indicators [4] and the Agency for Healthcare Research and Quality (AHRQ) Quality Indicators in the U.S. [5].

Social determinants have been widely acknowledged as the underlying causes of chronic diseases [6,7]. They refer to “the unequal conditions in which people are born, grow, live, work, and age; and the inequities in power, money, and resources that give rise to them” [6,8]. Area deprivation, one of the social determinants of health, refers to deteriorated and unfavorable living conditions for lack of material and social resources [9] and is independent of socioeconomic status at the individual level [10]. A systematic review found that patients with diabetes in the deprived areas are less likely to achieve glycemic control targets, have higher blood pressure and worse outcomes in lipid profile control [10]. A study in England found that more deprived areas had higher rates of inpatient admissions and readmissions necessitated by diabetes [11]. A study in Taiwan found that patients with diabetes who lived in areas with a low, medium, or high education level were more likely to undergo DRPH than those living in areas with the highest education level [12]. A recent study in South Korea found that type 2 diabetes patients with high socioeconomic deprivation had a higher hospital admission in areas with high socioeconomic deprivation than those in the low deprivation areas [13].

Access to resources is highlighted as a fundamental component of social determinants of health [14]. The United Nations underscored access to quality essential healthcare services as a core objective in achieving universal health coverage for *sustainable development goals* [15]. Since 2006, the Health Promotion Administration (HPA) of Taiwan has launched the Accredited Diabetes Health Promoting Institutes initiative to strengthen hardware equipment, enhance practical diabetes training of health care personnel, assure quality of care, raise awareness in health promotion for diabetes high risk population, and foster patient self-help groups in line with the Accreditation Scheme of Diabetic Shared Care Network and Diabetes Medical Benefits Improvement Project [16]. Two waves of nationwide surveys between 2006–2011 in accredited Diabetes Health Promotion Institutes in Taiwan showed that the percentages of subjects who had haemoglobin A1c lower than 7% (A) increased by 6.5%; both systolic and diastolic blood pressure lower than 130/80 mmHg (B) increased by 22%, and total cholesterol lower than 160 mg/dL or low density lipoprotein cholesterol lower than 100 mg/dL (C) increased by 57.8%, with a resulting total ABC attainment rate from 4.1% to 8.6% [17]. A further nationwide survey in 2018 showed that the goal achieving rate in A1C was at 42.2%, blood pressure at 37.3%, and LDL-C at 69.1%, and the final total ABC attainment rate was 12.4% [18], with improvement rates of 21.9% for A, −3.61% for B, and 24.0 for C compared with the results of the nationwide survey in 2011 [18]. Higher access to accredited Diabetes Health Promoting Clinics (DHPC) and Hospitals (DHPH) might contribute to the improvement of DRPH in the townships because patients could have better opportunities to receive quality diabetes care to avoid hospital admission in the living township with high densities of DHPC and DHPH.

Access to elderly social services (ESS) is an important social determinant apart from access to medical services and is conducive to the formation of social networks, further providing beneficial social determinants. A social network refers to the social relationships a person has during day-to-day interaction, which serves as the normal avenue for the exchange of opinion, information, and affection [7]. The role of the social network, among other things, is to suggest, advise, influence, or persuade an individual into taking or not taking particular courses of action [7]. Type 2 diabetes is prevalent among middle-aged and older adults in Taiwan [2]. The Taiwan government established a variety of ESS to encourage *active aging*, including community care centers, senior citizens learning centers, senior citizens learning camps, elderly welfare services centers, and evergreen academies. Social networks can influence diabetes self-management by assisting access to and promoting the use of resources, sharing knowledge in self-care, and engaging and maintaining productive relationships with network members [19,20]. A meta-analysis found that social networks could improve social support and hemoglobin A1c in 3 months [19]. Another literature review found increased diabetes self-management, medication adherence, adoption of nutritional and active lifestyles, and improved clinical outcomes with increased social support among patients with type 2 diabetes [21]. Higher access to ESS might have contributed to the improvement of DRPH in the townships because patients with diabetes could have better opportunities to establish a social network and receive social support for better self-management, medication adherence, and adoption of nutritional and active lifestyles to avoid hospitalization in the townships with high densities of ESS.

Little is known about the impact of geographical inequalities, notably area deprivation, DHPC/DHPH densities, and ESS densities on DRPH rates in townships across Taiwan. Understanding hot spots of DRPH rates and their social determinants is essential in prioritizing diabetes policy and geographical allocation of resources. This study aims to present the distributions of DRPH, area deprivation, DHPC and DHPH densities, and ESS density in all Taiwan townships. It further examines the associations of area deprivation, DHPC/DHPH densities, and ESS density with DRPH, with a focus on locally varying relationships, to show a vignette of the DRPH issue in Taiwan.

## 2. Materials and Methods

### 2.1. Data Sources

Data were obtained from the 2010–2016 2-million-patient sampling dataset of the National Health Insurance Research Database (NHIRD), 2010 Population and Housing Census [22], 2010 Income Tax Statistics [23], 2010–2016 Diabetes Health Promotion Institutes Roll Files [24], 2010–2016 Community Care Centers Files [25], 2010–2016 Elderly welfare services Center Files, 2010–2016 Evergreen Academy Files, 2010–2016 Senior Citizens Learning Center Files [26], and 2010–2016 Senior Citizens Learning Camps Files [27]. The NHIRD was maintained and released by the National Health Insurance Administration and Health and Welfare Data Science Center, Ministry of Health and Welfare (MHW). The NHIRD provides all inpatient and ambulatory medical claims of approximately 99% of Taiwanese people [28]. The accuracy of NHIRD was validated in previous studies [29]. The township was the unit of analysis. A total of 352 townships were included in this study, excluding those in Penghu County, Kinmen County, and Lienchiang County for the heterogeneous nature of those areas. This study was approved by the Research Ethics Committee of China Medical University Hospital (CMUH105-REC1-132).

### 2.2. Measurement

DRPH refers to the definition of hospitalization from the Prevention Quality Indicator (PQI) algorithm proposed by the Agency for Healthcare Research and Quality (AHRQ) and includes admissions for a principal diagnosis of diabetes short-term complications consisting ketoacidosis (ICD-9-CM 250.1 or their corresponding ICD-10-CM codes), hyperosmolarity (250.2), or coma (250.3), diabetes long-term complications (250.4), uncontrolled diabetes (250.0), and lower-extremity amputation (841.0) [5]. The DRPH rates were calculated as the aggregated number of discharges for DRPH among patients with diabetes aged 18 and above in a township between 2010 and 2016 divided by the aggregated number of those with diabetes aged 18 and above between 2010 and 2016 (person-year) in a township multiplied by 1000. The included patients with diabetes must have at least 2 outpatient visits or 1 inpatient hospitalization with primary diagnoses of diabetes (ICD-9-CM: 250.xx or A181 and ICD-10-CM: E08-E13) each year during 2010 and 2016. We excluded diabetes patients with any primary diagnosis of complications of pregnancy, childbirth, and the puerperium (ICD-9-CM: 630-676 or A38-A41 and ICD-10-CM: O00-O9A).

Area deprivation index was constructed using principal component analysis of 5 socioeconomic variables―median household income; proportion of people aged above 6 and either at school or holding a paid job; proportion of individuals without a diploma from senior high schools or senior vocational high schools; proportion of individuals employed in agriculture, forestry, fishery and animal husbandry; and proportion of individuals with divorce, separation, or a demised spouse in townships. The factor loadings of each of the five variables were 0.76, 0.64, 0.96, 0.73, and 0.86, respectively. The index accounted for 64% of the variability in the included variables. The higher the score, the more deprived it is. Average ESS density (per km^2^) was calculated as the average number of ESS institutions between 2010–2016 in a township divided by the township area. The social services institutions included community care centers, senior citizens learning centers, senior citizens learning camps, elderly welfare service centers, and evergreen academies. Average DMHP hospital/clinic density (per km^2^) was calculated as the average number of DMHP hospitals/clinics between 2010 and 2016 in a township divided by the township area. Moreover, demographic and health characteristics included average percent population aged ≥ 65 between 2010–2016 in townships and diabetes complications severity which was based on adjusted diabetes complications severity index (aDCSI) [30,31] and calculated as the average aDCSI scores among patients with diabetes between 2010–2016 in townships.

### 2.3. Geographical Incidence Estimation

Although the comprehensive medical visit information is recorded in the NHRID, the townships where the patients lived are not registered in compliance with *the Personal Information Protection Act*. Previous studies had to rely on the location of the insurance unit where the patient registered to analyze health disparity by geographic location. A study found that the equivalence between the insured and current residence is very low at only 39.6% for the district-township level [32]. We further referred to a previous study that identified residence by using the commuter time matrix to compare location proximity among permanent residence and different locations of access to healthcare for upper respiratory infections (ICD-9-CM: 460–466, 487 and the corresponding ICD-10-CM codes), in clinics or in hospital ambulance in a year; that study showed that the equivalence between the insured and current residence was up to 81.2% at the district-township level [32].

### 2.4. Statistical Analysis

Descriptive data analyses were conducted for township-level environmental, demographic, and health characteristics. Pearson’s correlations and bivariate associations were tested between all township-level covariates and the primary outcome, DRPH rate. Gender ratio was excluded due to a high correlation with area deprivation (r = 0.79). Ordinary least squares (OLS) regression was used to investigate factors associated with the DRPH rate, with the township as the unit of analysis. Descriptive statistics, Person’s correlation analysis, and unadjusted OLS regression were conducted using SPSS 25.0 (IBM Corp., Armonk, NY, USA). Two-sided tests with *p* < 0.05 were regarded as the statistical significance and the global multicollinearity was assessed according to the variance inflation factors (VIF). In the multiple OLS model, the values of VIF were between 1.05 and 1.79, much lower than the recommended threshold value of 10 for multicollinearity [33]. In the GWR model, the values of the condition number field were less than 30, indicating that the results were stable and reliable due to a lack of strong local multicollinearity [34].

In spatial analysis, we mapped the variable distributions with natural break classification, a method for best grouping similar values together and distinguishing the differences between classes [35]. Furthermore, Anselin local Moran’s I with inverse distance methods with row standardization was conducted to examine spatial outliers of area deprivation indexes, ESS densities, DHPC densities, and the DRPH rates [36]. Global Moran’s I test was then conducted to examine spatial autocorrelation of residuals in the OLS and geographically weighted regression (GWR) models; a higher positive Moran’s I approaching 1 indicates that values in the neighboring areas tend to cluster, whereas a lower negative Moran’s I approaching -1 suggests that values are dispersed [36]. Finally, GWR was conducted to handle residual spatial autocorrelation in the OLS model and allowed parameter estimates to vary over space [36,37].

The adaptive kernel, a function of a specified number of neighbors and appropriate for the distribution of observations varying across space, was used [38,39]. In our case, observations (townships) are much smaller and closer together in the West than they are in the East areas. The coefficients of each independent variable and adjusted R^2^ and corrected Akaike information criterion (AICc) in both OLS and GWR were summarized. The local t-values for the primary parameter of area deprivation index, ESS density, DHPC density were calculated by dividing the coefficient estimate by its standard error for creating a surface of estimated coefficients [37,40]. The critical value for t-value is 1.96. Spatial analyses were conducted using ArcGIS, version 10.5.

## 3. Results

Table 1 presents the results of the descriptive and bivariate analyses. The DRPH rates ranged from a low level of zero discharges per 1000 person-years in Laiyi Township, Pingtung County to a high level of 104 discharges per 1000 person-years in Taoyuan District, Kaohsiung City. The average was 33.05 discharges per 1000 person-years. The DRPH rates were significantly and positively associated with area deprivation, percent population aged ≥ 65, and aDCSI scores. By contrast, the DRPH rates were significantly and negatively associated with the ESS density and DHPC density. The gender ratio of the patients with diabetes in the townships was 106.81 males per 100 females; the patients had 1.14 aDCSI scores (SD 0.19) and 1.06 Charlson Comorbidity Index score (SD 0.19) on average.

**Figure 1:**
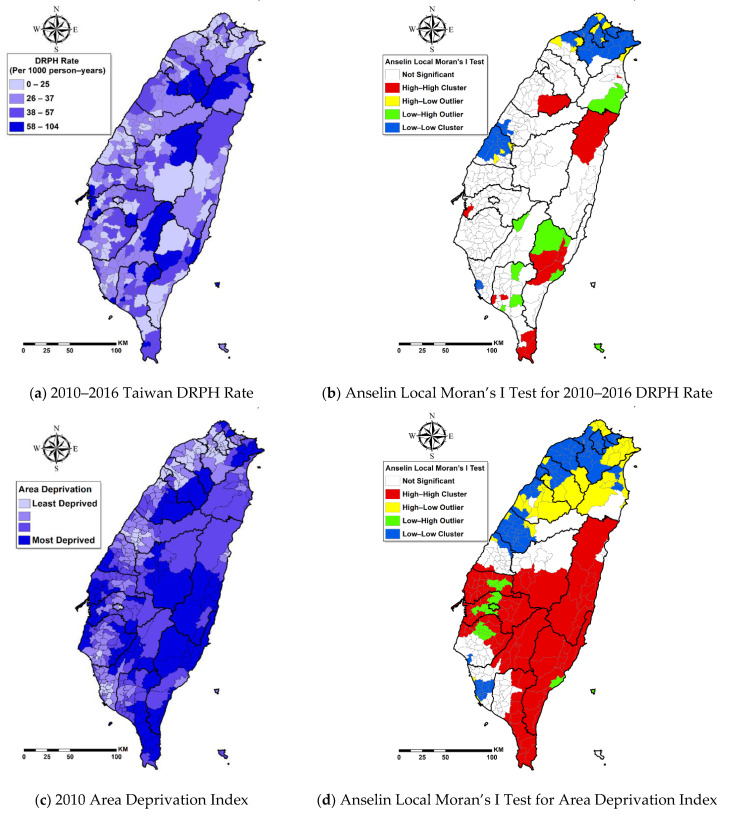

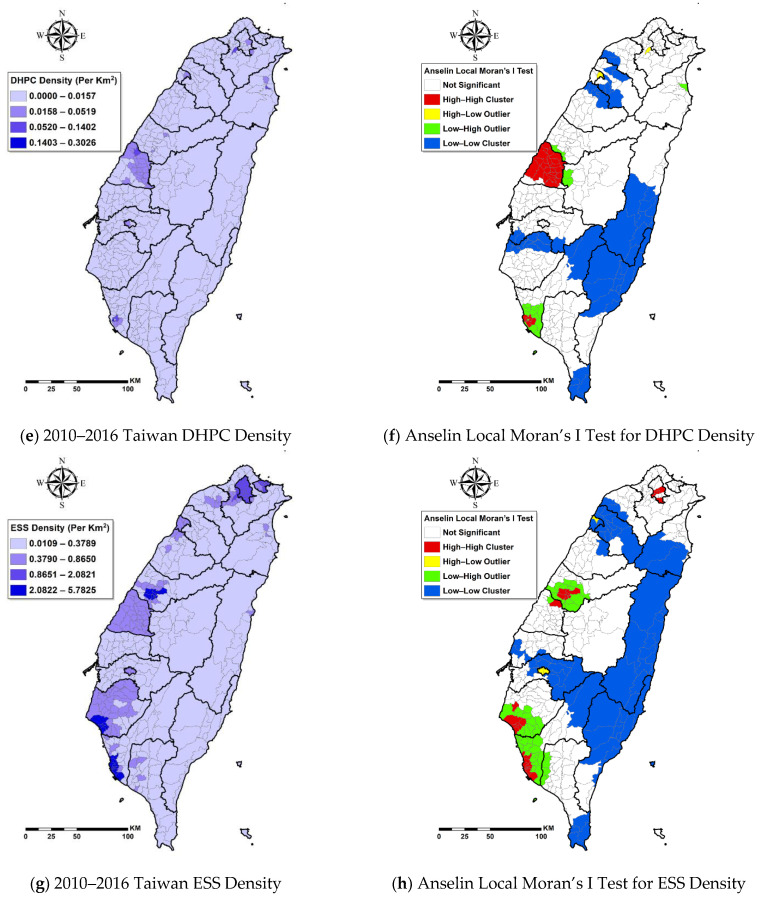
Maps highlighting geographical variations in key variables and local spatial autocorrelation results. Note: DRPH, diabetes-related preventable hospitalization; DHPC, diabetes health promoting clinics; ESS, elderly social services.

Figure 1a,c,e,g presents the geographic distribution of DRPH rates, area deprivation indexes, DHPC densities, and ESS densities, respectively. The townships with the highest DRPH rates were located in the Central, Mid-north, and Southeast areas. The Upper-Midwest, Central, Southeast, Southwest, and South townships had socioeconomic deprivation. The Southwest and Midwest townships had the highest DHPC densities. The Southwest, Midwest, and North townships had the highest ESS densities. Based on Anselin Local Moran’s I Test, low–low clustering for DRPH rates was located in the North and Midwest townships (Figure 1b). High–high clustering for socioeconomic deprivation was located in the Central and Southeast townships, whereas low–low clustering was located in the Northwest townships (Figure 1d). High–high clustering for DHPC densities was located in the West Central, whereas low–low clustering was located in the Southeast townships (Figure 1f). Low–low clustering for ESS Densities was located in the East, Upper Central, and Central Under townships Figure 1h.

In the OLS results Table 2, we found that townships with greater socioeconomic deprivation and higher aDCSI scores had higher DRPH rates, whereas those with higher ESS densities and DHPC densities had lower DRPH rates. The OLS model explained 21.38% of variation. We further tested the extent of spatial autocorrelation and examined the residuals from the OLS model and found that Global Moran’s I was statistically significant (z-score = 3.983, *p* < 0.001). This result indicated a spatial autocorrelation.

**Figure 2:**
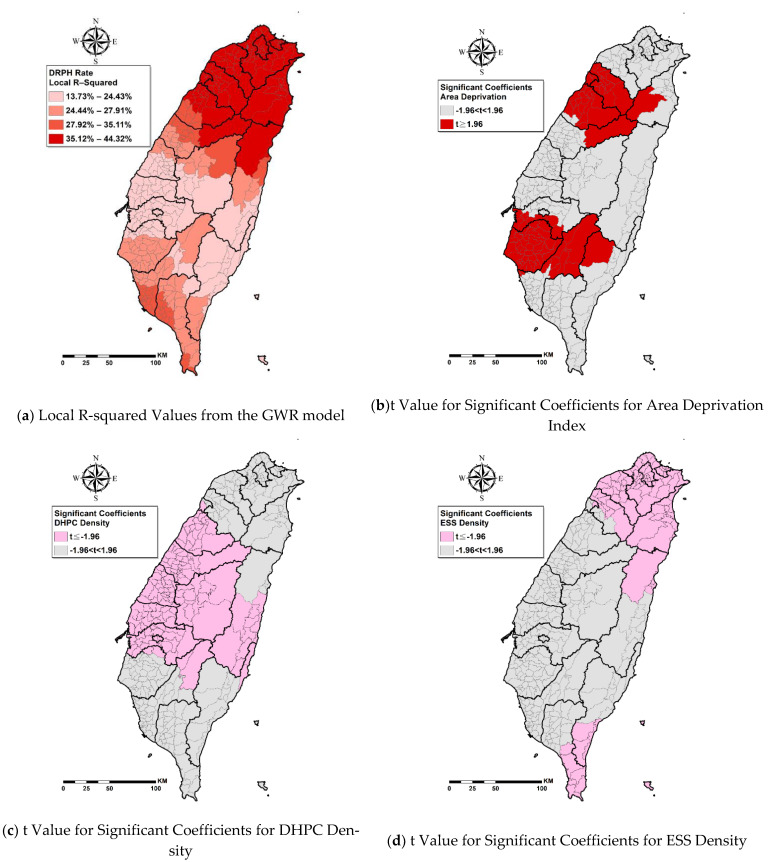
Maps for geographically weighted regression (GWR) results.

The GWR model had a higher overall adjusted R^2^ value (28.37%) than that of the OLS Model (21.38%), suggesting a better explanation of DRPH rates by the GWR model. The adjusted R^2^ for the local GWR model ranged from 13.73% to 44.32% (96.30% of townships had improved R^2^ values compared with the OLS Model) (Figure 2a), with a true relative percentage 32.69% and absolute percentage 6.99% improvements over the OLS Model. The AICc in the GWR model (2807.02) was slightly smaller than that in the OLS model (2822.05), indicating a better fit. Figure 2b–d demonstrates the spatial relationships of DRPH rate with area deprivation, DHPC density, and ESS density by statistically significant coefficients, adjusted by the covariates. The GWR model indicated that 90 townships (25.6%) had significantly positive correlations between area deprivation and DRPH rates (Figure 2b), 142 townships (40.3%) had significant negative correlations between DHPC density and DRPH rates (Figure 2c), and 100 townships (28.4%) had significant negative correlations between ESS density and DRPH rates (Figure 2d). Moreover, the GWR model results also indicated that 293 townships (83.20%) had significant positive correlations between aDCSI scores and DRPH rates, while 83 townships (23.60%) had significantly positive correlations between diabetes prevalence scores and DRPH rates (Table 3). Global Moran’s I test for spatial autocorrelation on the GWR residuals showed a statistically insignificant z-score (z = −0.23, *p* = 0.821), indicating no significant spatial autocorrelation.

## 4. Discussion

To the best of our knowledge, this study is the first research using spatial analysis to examine the associations of DRPH rates with area-level socioeconomic deprivation as well as densities of the DHPC and ESS. In particular, the role of access to the accredited diabetes health care in clinics/hospitals and ESS densities have been rarely discussed in the context of preventing avoidable hospital admissions among patients with diabetes. Spatial analysis revealed geographical inequality in DRPH rates, area deprivation, the DHPC densities, and ESS densities. Analysis based on GWR showed that higher DRPH rates are significantly related to lower DHPC densities, lower ESS densities, and higher socioeconomic deprivation, and the relationships were spatially nonstationary across Taiwan. In the global OLS model, 21.38% of township-level diabetes prevalence was explained by area deprivation, DHPC densities, ESS densities, and aDCSI score. The GWR model had a better explanation of DRPH rates (28.37%). At an individual township level, the explanatory percentage ranged from 13.73% to 44.32%, and 96.30% of townships had improved the R^2^ values than those of the OLS Model. The GWR model presented a better fit because its AICc is smaller than that in the OLS model [34].

This study strived to fill the knowledge gap of geographical inequality of DRPH and their relationships with access to resources for patients with diabetes at the township level, which has rarely been discussed in previous research. In Taiwan, previous studies focused on the risk of DRPH among individual patients with diabetes and identified influencing factors largely at the individual level and only a few at the institutional and township levels. At the individual level, patients with diabetes were more likely to undergo DRPH if they were male [41,42], aged 65 and above [41,42,43], had low individual/household incomes [12,41,42,43], not taking regular physical activities [44], with higher comorbidities [12,42,43,44] and DCSI scores [41,43], had hospital admissions [43] and physician visits in the previous years [41], not enrolled in the pay-for-performance program [12,42], and the care had low continuity and coordination [41]. At the institutional and township levels, patients with diabetes were more likely to undergo DRPH if they received treatments in hospitals than in clinics [12], lived in the townships with lower coverage of higher education [12] and less physician density [43], lived in rural areas [41,43] or southern area than the capital city of Taipei [42]. However, the above findings fell short in informing prioritization of diabetes policies and geographical allocation of resources for townships.

These results showed that greater area deprivation is related to a higher DRPH rate, which is consistent with previous studies in England [11] and Germany [45] but contradicted a previous study in Western Victoria, Australia [46]. Furthermore, no investigation has looked into the non-stationarity of these relationships. This study found no consistent associations between area deprivation and DRPH across Taiwan in the GWR model, but it further identified significantly positive relationships only in some townships of Hsinchu City, Hsinchu County, Miaoli County, Chiayi County, and Tainan City (Figure 2b). Deprived areas are commonly related to less collective material and social resources such as recreational and/or health services, job opportunities, and social support [47]. A systematic review found that the development of type 2 diabetes was related to fewer health services, facilities for physical activities, and amenities for healthy food [48]. A German ecological study found that higher hospitalizations for diabetes are related to socioeconomic factors such as higher unemployment rates and lower percent of the labor force with academic degrees [45]. A previous study in Taiwan found that patients with diabetes who live in the township with lower coverage of higher education had higher risks of DRPH [12]. Previous studies in Taiwan also found that diabetic patients in low- and middle-income brackets tended to have DRPH relative to those with higher incomes [12], and the hospitalized group tended to have lower educational status than those patients in the never-hospitalized group [44]. Considering that individual socioeconomic status is generally correlated to the regional socioeconomic status, the proportion of patients with low individual SES is probably proportionally higher in the more deprived regions with less education resources and employment opportunities. Deprived areas are similar to rural areas in terms of low accessibility to health services. Another study in Taiwan found that rural diabetic patients had fewer chances of receiving guideline-recommended examinations and had more chances of having diabetes-related hospitalization [43]. In Taiwan, accessibility barriers persist that can make access to appropriate health care service difficult for individuals in certain areas [49]. The “Health in All Policies (HiAP)” is regarded as an effective approach to promoting population health [50]. Policymakers at the national, city, and community levels should pay extra attention to deprived townships highlighted in Figure 1b,c to put health on the agenda in all sectors and allocate health-orientated material and social resources to reduce socioeconomic inequality for improving DRPH.

Our results showed that higher certificated DHPC densities are significantly associated with lower DRPH rates and highlighted the importance of this relationship particularly in Central Taiwan (Figure 2c). The implementation of certifiable management systems, by strengthening the structure of health organizations and processes of service delivery, is considered a strategy to improve the quality of care. Previous studies showed such a system―the Accredited Diabetes Health Promoting Institutes initiative―improved quality outcome of diabetes care [17,18]. However, little is known about the impact of accessibility to certificated high-quality diabetes care facilities on preventable hospitalization, and the non-stationarity of these relationships at the area level. The Accredited Diabetes Health Promoting Institutes include clinics and hospitals. In Taiwan, outpatient services in hospitals accounted for 25.27% of the national health expenditure and 22.89% in clinics [51]. Patients with diabetes could receive diabetes care in clinics or the outpatient department in hospitals. This study highlighted the importance of access to certificated DHPC for high-quality diabetes care in preventing avoidable hospital admissions. In Taiwan, DHPC clusters in Changhua County (Figure 1f) where significantly lower DRPH rates were observed (Figure 2f). DHPC is organized as interdisciplinary care teams which constitute a barrier for small-scale-practices in primary care clinics [52]. The Health Bureau of Changhua County treated all of the clinics in a given community as a unit that shared the workforce and costs to attain adequate scale of economy to compensate for the shortage of professionals in the remote areas by coordinating the recruitment of dieticians and deploying outreach eye-care clinics [52]. Changhua County can be an example for other cities/counties to facilitate their clinics to strengthen the structure and improve the process of diabetes care and to apply for certification for quality improvement. The other cluster of DHPC was found in some townships in Kaohsiung. However, it did not significantly contribute to the reduction in DRPH. In those townships, the less severe diabetes complications appeared to have a desirable effect on DRPH. This finding is encouraging because, in the long run, diabetes complication severity of patients can be reduced through certification of diabetes care due to enhancement of structure, process, and outcome of diabetes care.

Furthermore, this study found that higher ESS densities are significantly related to lower DRPH rates, and it highlighted the importance of this relationship particularly in Northern, Northeast, and Southern Taiwan (Figure 2d). This study filled the knowledge gap regarding the associations between ESS densities and DRPH rates and the non-stationarity of these relationships at the area level. Previous studies found that social networks and social support may improve self-management, medication adherence, and adoption of nutritional and active lifestyles among patients with diabetes [19,20,21]. Social networks and social support hinge on social participation which can be enhanced through improved accessibility of an age-friendly environment and essential social services in the neighborhood to promote health and wellbeing. In 2002, the World Health Organization (WHO) proposed a policy framework of active aging and defined active aging as “the process of optimizing opportunities for health, participation, and security in order to enhance the quality of life as people age” [53] (p.12). In this framework, social environments are identified as important factors of active aging, including social support, education, and literacy. One year later, the European Centre for Social Welfare Policy Research cooperated with the United Nations Economic Commission for Europe to develop the Active Aging Index which emphasizes participation in society, healthy living, and capacity and enabling environment for active aging [54]. In 2007, WHO proposed a guide for global age-friendly cities which underscores the importance of social participation through accessible and various opportunities [55]. Therefore, the accessibility and opportunity of social participation for social network and social support become very important. Patients with newly diagnosed diabetes in Taiwan were 61 years old on average with the standard deviation of 10 years [56], suggesting a substantial need for elderly social services. This study chimed harmoniously with the above-mentioned advocacies and demonstrated the importance of ESS densities on DRPH rates. Following this result, we urge policymakers from the Ministry of Education and MHW to further invest in social services in Northern, Northeast, and Southern Taiwan with low accessibility to ESS Figure 1d,g,h.

Some limitations of our study should be noted. First, this research is a cross-sectional study and cannot be used to infer causality, although we used 7-year data to stabilize the relationships. Second, we conducted GWR with adaptive kernel style which accounted for differences in the size of townships and their distance of influence. However, GWR is inevitably limited by the edge effect, whereby townships located on the edges of Taiwan (i.e., coastal townships) have no 360° influence of townships in the country’s interior. Third, GWR cannot respond to spatial spillover effects. The spillover effect commonly occurs on socioeconomic conditions and healthcare resources across neighboring areas. Future studies may consider further employing the Spatial Durbin model to estimate the direct and spillover effect of the change in the township characteristics on the DRPH [57,58,59]. Lastly, the overall adjusted R^2^ value from GWR accounted for 28.37% of township-level DRPH rates which is less than one-third of the variance; whereas it is higher than that of other studies regarding influencing factors of other disease prevalence or mortalities in Taiwan [60,61,62]; room for improvement remains in our modeling. The local R^2^ values ranged from 13.73% to 44.32% of township-level DRPH rates. The seven variables included in the model explained less than one-fifth of the variance in DRPH rates in 11 townships particularly clustering in Hualien County and Taichung County, which means some of the relevant factors related to DRPH rates in these townships have been missing from our model. The “AICs values” for the OLS and GWR models are relatively high, whereas the “Adjusted R^2^” is relatively low. The “Adjusted R^2^” for both OLS and GWR models are lower than 30%, suggesting that alternative methods must be adopted in future research to improve goodness of fit.

## 5. Conclusions

This study found that the DRPH rates are high in townships of Taiwan that had greater socioeconomic deprivation and lower densities of certificated DHPC and ESS, and such associations spatially varied in magnitudes. Diabetes care policies can be tailored locally to meet local health needs. This study highlights the difference in resources for patients with diabetes determining the DRPH rates across Taiwan and subsequently identifies hot spots for inadequate DHPC. Policymakers at national and local levels should move from working in silos to adopt an intersectoral and holistic approach of HiAP to prevent DRPH through additional and better-integrated resources for essential quality diabetes care in clinics, in addition to facilitating socioeconomic development and encouraging elderly social participation in the townships.

## Figures and Tables

**Table 1:** A profile of the township characteristics and bivariate analysis.

Variable	Minimum	Maximum	Mean (SD)	Coefficient ^1,2^
Dependent variable				
DRPH rate (per 1000 person-years), 2010–2016 ^3^	0	104.20	33.05 (14.81)	
Environment Factors				
Area deprivation Index, 2010	−2.56	2.72	0.00 (1.00)	4.42 ***
DHPC density (per km^2^), 2010–2016 ^5^	0	0.30	0.01(0.02)	−99.37 **
DHPH density (per km^2^), 2010–2016 ^6^	0	1.62	0.02 (0.10)	−3.18
ESS density, 2010–2016 ^4^	0.01	5.78	0.66 (1.07)	−2.97 ***
Demographic and Health characteristics				
Percent population aged ≥ 65, 2010–2016	0.06	0.27	0.14 (0.04)	51.67 **
aDCSI score, 2010–2016 ^7^	0.66	1.84	1.14 (0.19)	30.59 ***
Diabetes prevalence (per 1000), 2010–2016	2.61	79.02	29.72 (7.66)	−0.09

^1^ Boldface estimates indicate statistical significance (** *p* < 0.01; *** *p* < 0.001); ^2^ Parameter estimate for simple linear regression; ^3^ Diabetes-Related Preventable Hospitalization; ^4^ Elderly social service; ^5^ Diabetes health promoting clinics; ^6^ Diabetes health promoting hospitals; ^7^ Adjusted diabetes complications severity index.

**Table 2:** Township-level ordinary least squares regression results.

Variable	Parameter Estimate	SE	*p*-Value
Area deprivation, 2010	2.96	0.94	0.002
DHPC density, 2010–2016	−66.36	30.12	0.029
DHPH density, 2010–2016	13.00	8.28	0.117
ESS density, 2010–2016	−1.85	0.83	0.027
Percent population aged ≥ 65, 2010–2016	−18.34	20.43	0.370
aDCSI score, 2010–2016	26.98	3.96	<0.001
Diabetes prevalence (per 1000), 2010–2016	−0.12	0.09	0.207
AICc ^1^	2822.05		
Adjusted R^2^	21.38%		

^1^ Corrected Akaike information criterion.

**Table 3:** Township-level geographically weighted regression results.

Variable	Coefficient (β)				Percentage of Township by 95% of t-Statistic		
	Min	Median	Max	Mean (SD)	t ≤ 1.96	−1.96 < t < 1.96	t ≥ 1.96
Area deprivation	0.22	2.48	5.75	2.88 (1.28)	0%	74.40%	25.60%
DHPC density, 2010–2016	−494.18	−62.70	−27.61	−127.22 (100.25)	40.30%	59.70%	0%
DHPH density, 2010–2016	−83.51	15.52	20.48	8.53 (12.94)	1.40%	98.60%	0%
ESS density, 2010–2016	−4.58	−1.77	6.29	−2.15 (1.25)	28.40%	71.60%	0%
Percent population aged ≥ 65,	−91.03	−14.81	53.73	−19.45 (37.20)	7.10%	92.90%	0%
2010–2016							
aDCSI score, 2010–2016	9.22	28.36	44.1	27.24 (10.28)	0%	16.80%	83.20%
Diabetes prevalence (per 1000),	−0.71	−0.19	0.06	−0.23 (0.23)	23.60%	76.40%	0%
2010–2016							
AICc	2807.02						
Adjusted R^2^	28.37%						

## Data Availability

Data sharing is not applicable.
